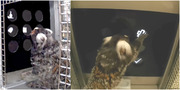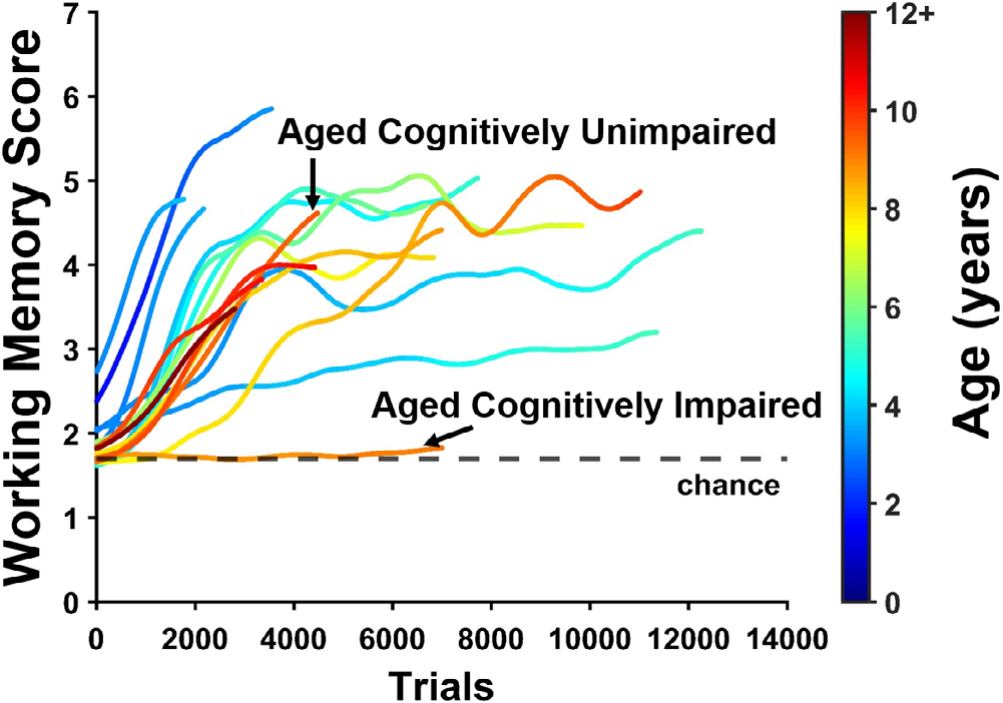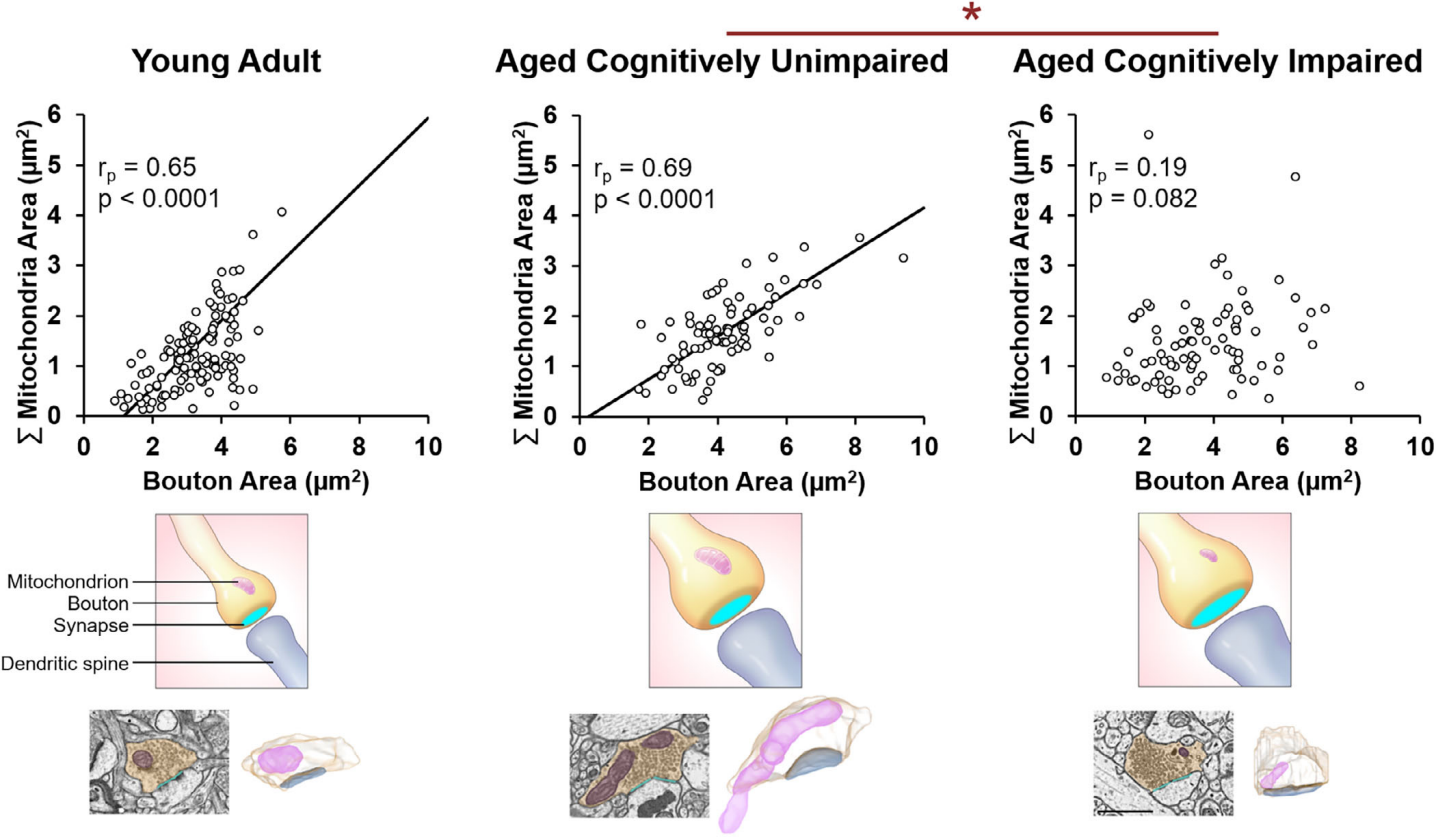# Marmoset cognitive aging: Heterogeneity and synaptic dysfunction

**DOI:** 10.1002/alz.085459

**Published:** 2025-01-03

**Authors:** Courtney Glavis‐Bloom, Casey R Vanderlip, Payton A Asch, Sammy P Weiser Novak, Masaaki Kuwajima, Lyndsey M Kirk, Kristen M Harris, Uri Manor, John H Reynolds

**Affiliations:** ^1^ Salk Institute for Biological Studies, La Jolla, CA USA; ^2^ University of Texas at Austin, Austin, TX USA

## Abstract

**Background:**

As humans age, some experience cognitive impairment while others do not. When impairment occurs, it varies in severity across individuals. Translationally relevant models are critical for understanding the neurobiological drivers of this variability, which is essential to uncovering the mechanisms underlying the brain’s susceptibility to aging. The common marmoset has emerged as an advantageous non‐human primate model to investigate the biological consequences of aging due to shared behavioral, neuroanatomical, and age‐related neuropathological features with humans, and a short lifespan that facilitates longitudinal studies. Despite growing popularity as a model, robust cognitive phenotyping of marmosets, particularly as a function of age and across multiple cognitive domains, is lacking.

**Method:**

To address this major limitation for the development and evaluation of the marmoset as a model of cognitive aging, we developed a comprehensive touchscreen‐based neuropsychological test battery. We use this battery to longitudinally assess cognitive aging trajectories in marmosets across the lifespan. To characterize synaptic ultrastructure as a function of aging and cognitive status, we used electron microscopy.

**Result:**

Similar to humans, we find that marmosets show age‐related impairment in motor speed, motivation, cognitive flexibility, and working memory, with a remarkable degree of heterogeneity. We also find that aged marmosets show synapse loss in the dorsolateral prefrontal cortex at a level consistent with aged macaques. Though synapse loss is widely considered a key determinant of cognitive impairment with age, we find an equivalent degree of synapse loss in aged cognitively unimpaired and aged cognitively impaired marmosets. Our findings indicate that the coordinated scaling of the sizes of synapses and mitochondria with associated boutons is crucial for preserving working memory with age. However, failure of synaptic mitochondria to scale with presynaptic boutons selectively underlies age‐related working memory impairment.

**Conclusion:**

We posit that failed scaling results in mismatched energy supply and demand, leading to impaired synaptic transmission. This novel mechanism of synapse dysfunction differentiates aged marmosets with cognitive impairment from those without. Together, this work demonstrates the importance of comprehensive cognitive phenotyping to uncover the neurobiological consequences of aging, and firmly establishes the common marmoset as an advantageous model for age‐related cognitive impairment.